# Prognostic analysis of children with tetralogy of Fallot through a small incision in the right axilla

**DOI:** 10.3389/fcvm.2025.1457554

**Published:** 2025-06-30

**Authors:** Fanwei Meng, Jianchao Li, Weijie Liang, Haoju Dong, Bing Li

**Affiliations:** Fuwai Central China Cardiovascular Hospital, Henan Provincial People’s Hospital, Zhengzhou, Henan, China

**Keywords:** minimally invasive surgery, tetralogy of Fallot, right subaxillary vertical thoracotomy, cardiopulmonary bypass, median sternotomy incision

## Abstract

**Objective:**

Compare the clinical efficacy of a minimally invasive small incision in the right axilla vs. traditional median sternotomy in the surgical treatment of tetralogy of Fallot (TOF).

**Methods:**

A retrospective analysis was conducted on 330 infants and young children under the age of 3 who underwent radical surgery for tetralogy of Fallot between March 2022 and March 2024. Patients were categorized into two groups based on the surgical approach. To ensure the consistency of preoperative baseline data (weight, gender, age, O_2_ saturation, main pulmonary artery and pulmonary branches diameter, McGoon ratio) between the two groups, the propensity score matching method was applied for 1:1 matching, resulting in two cohorts of 228 cases. The minimally invasive group (*n* = 114) received surgery through a small incision in the right axilla, while the median sternotomy group (*n* = 114) underwent surgery via median sternotomy. Clinical parameters including demographic data (weight, gender, age, O_2_ saturation, main pulmonary artery and pulmonary branches diameter, McGoon ratio), cardiopulmonary bypass metrics (duration of bypass, aortic cross-clamp time), duration of mechanical ventilation, intensive care unit (ICU) stay, postoperative chest drainage volume within 24 h, pulmonary valve regurgitation, and complications (reintubation, peritoneal dialysis, reoperation, extracorporeal membrane oxygenation (ECMO) use, infection, and mortality) were collected for comparison between groups.

**Results:**

No statistically significant differences were observed between the two groups in 24 h chest drainage volume, mortality, reintubation, reoperation, ECMO use, and infection. However, the minimally invasive group showed significantly shorter ventilator duration and ICU stay and a reduced rate of peritoneal dialysis (all *p* < 0.05).

**Conclusion:**

In infants and children under 3 years old with TOF, surgical correction via a right axillary small incision achieves equivalent clinical outcomes to traditional median sternotomy, without increasing postoperative mortality or complication rates. In addition, the minimally invasive approach offers benefits of reduced surgical trauma and enhanced postoperative recovery.

## Introduction

Lateral thoracotomy cardiac surgery has gained widespread adoption in adult coronary artery bypass grafting and valve procedures due to its superior reduced trauma and faster recovery rates compared with traditional median sternotomy (1–3). In small-incision approaches, extracorporeal circulation is conventionally established via femoral vessels to optimize surgical exposure. However, pediatric populations face unique challenges: smaller femoral vessel diameter increases the risks of cannulation-related complications, compromises extracorporeal flow rates, and raises intraoperative safety concerns. Consequently, pediatric minimally invasive cardiac surgery preferentially employs right axillary incision access for extracorporeal circulation—a methodology well established in correcting simple atrial septal or ventricular septal defects (VSDs) (4–6). The evolution of minimally invasive tetralogy of Fallot (TOF) correction has prioritized trauma reduction alongside long-term efficacy. While prognostic analyses confirm satisfactory survival and quality of life, persistent risks of reintervention and arrhythmias demand attention. As the most common cyanotic congenital heart disease, TOF has witnessed growing interest in minimally invasive correction. Notably, small right axillary incision approaches achieve both anatomically sound repair and enhanced cosmetic results (7). Critical technical considerations, particularly valve-sparing repair vs. transannular patch techniques, profoundly impact outcomes and require individualized assessment (8). Since 2020, our institution has pioneered this approach for TOF in infants and young children, and this report summarizes our technical experience.

## Material and methods

### Study design

**Figure 1:**
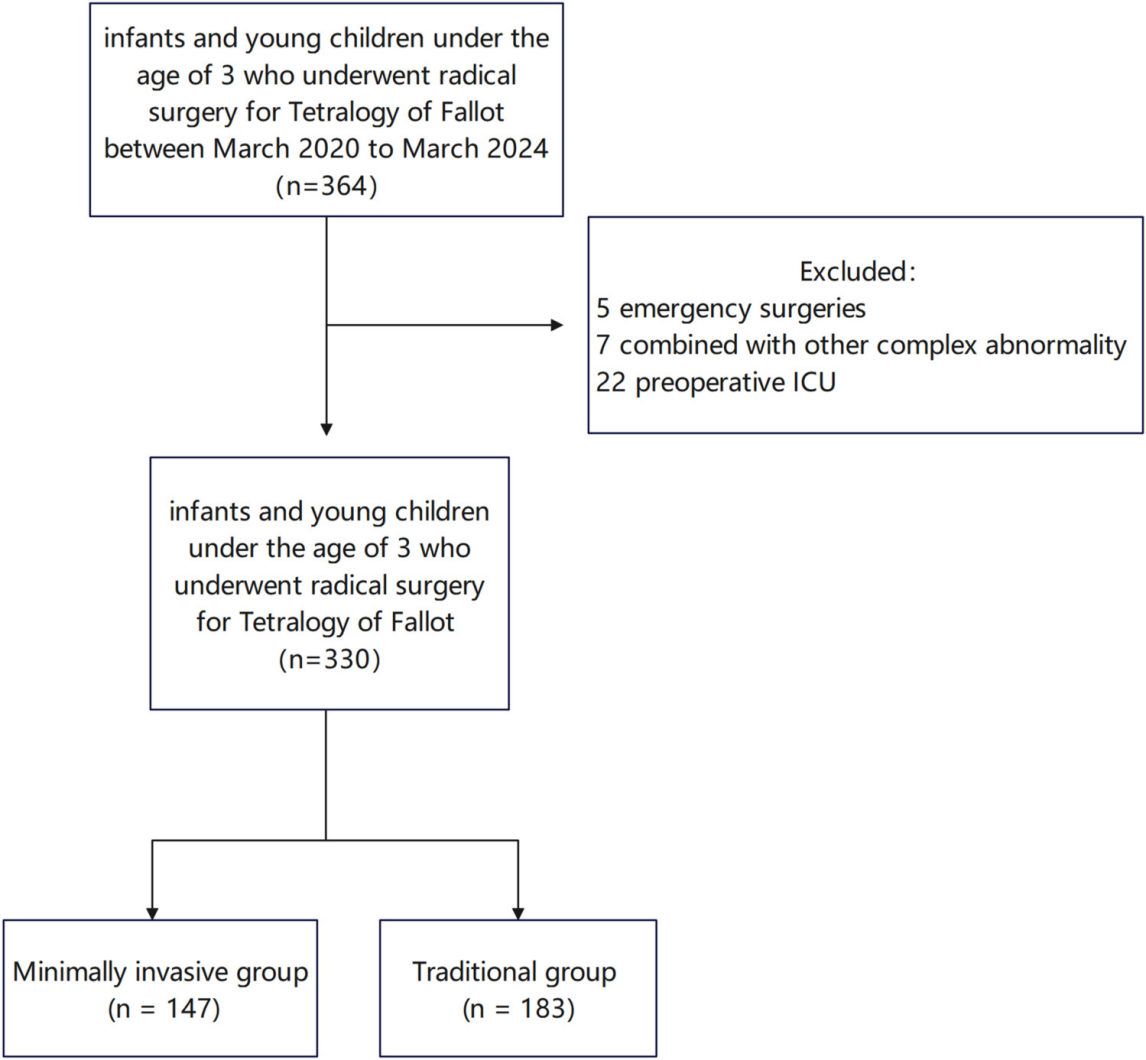
Outcomes of infants and young children under the age of 3 who underwent radical surgery for tetralogy of Fallot.

We conducted a retrospective, single-center study by reviewing the medical records of all infants and young children under 3 years of age who underwent radical surgery for tetralogy of Fallot between March 2022 and March 2024 at the Children's Heart Center of Fuwai Central China Cardiovascular Hospital as shown in Figure 1. Internal review board approval was obtained for a retrospective chart review (Fuwai Central China Cardiovascular Hospital Ethics Approval: 2023–57). Exclusion criteria were as follows: (1) patients admitted to the ICU preoperatively; (2) presence of other complex congenital heart diseases, such as pulmonary venous ectopic drainage, and endocardial cushion defects; and (3) emergency surgical cases. Primary objective: to evaluate the early postoperative outcomes (mortality, incidence of complications, etc.) of TOF repair surgery. Secondary objectives: to evaluate term functional outcomes and quality of life and to compare with conventional techniques (if applicable).

### Surgical procedure

**Figure 2:**
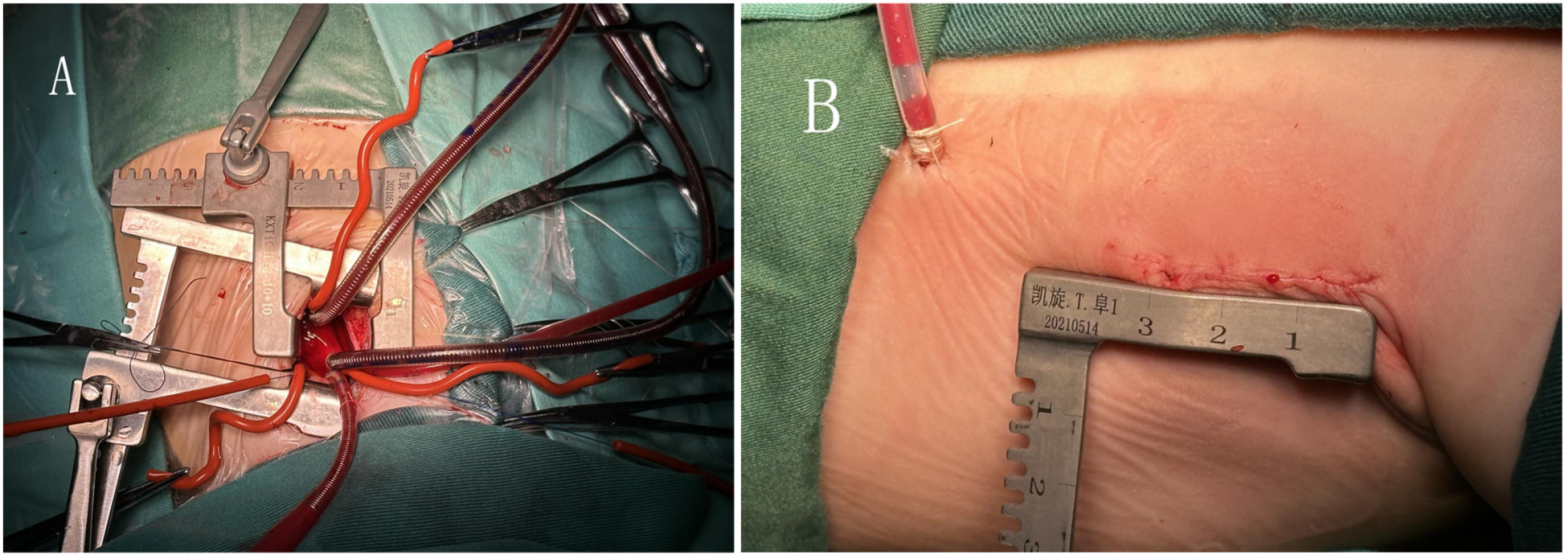
(**A**) Extracorporeal circulation catheterization through a small incision under the right axilla. (**B**) The length of the small incision under the right axilla.

All patients received tracheal intubation combined with intravenous anesthesia. In the minimally invasive group, infants and children were positioned in the left lateral decubitus position. A right lateral thoracic incision of approximately 3 cm was made between the mid-axillary line and the fourth rib. The median sternotomy group underwent thoracotomy via a median sternotomy. In both groups, cardiopulmonary bypass was established by cannulating the ascending aorta as well as the superior and inferior vena cava (as shown in Figure 2). The ascending aorta intubation (Medtronic, DLP, or Ningbo Feral Medical, China) and superior and inferior vena cava intubation (Changzhou Longlaifu Medical Technology Co., China) in the minimally invasive group were two models smaller than the median sternotomy group. The cannulation details for cardiopulmonary bypass in the two groups are shown in Table 1. Children in the minimally invasive group needed negative pressure–assisted venous drainage during cardiopulmonary bypass, and the negative pressure value was maintained at −20 to −40 mmHg. The right lung was protected with wet gauze, and the pericardium was suspended. Depending on the presence of pulmonary valve annular stenosis and the degree of pulmonary artery development, a decision is made regarding whether to perform a transannular patch repair or extension to the main pulmonary artery or distal pulmonary branches. Following cardiac arrest, the hypertrophic muscles of the right ventricular outflow tract (RVOT) were resected through an infundibular ventriculotomy. Two retractors were then placed on each side of the ventriculotomy to fully expose the defect. The transatrial approach was the preferred method for VSD repair. The VSD was repaired using a continuous suture technique with a Dacron patch. After complete resection of the hypertrophic infundibular muscles, dilators were used to assess the diameters of the RVOT and pulmonary annulus to determine the feasibility of simple commissurotomy. Our fundamental principle was to preserve pulmonary valve function whenever possible and avoid transannular patching, as it may adversely affect long-term outcomes in TOF patients. If the measured diameter was more than 2 SDs below the weight-adjusted standard value, further evaluation of the number of leaflets, pliability, and commissural fusion was performed. When incision of fused commissures and improvement of pliability still failed to achieve an adequate pulmonary annulus, the annulus and main pulmonary artery were longitudinally incised, with distal extension if necessary. RVOT and pulmonary artery augmentation were performed using an autologous pericardial patch. Immediately after weaning from cardiopulmonary bypass, the flow velocity across the RVOT and supravalvular pulmonary artery was measured, with velocities below 3 m/s considered acceptable. Among the 330 infants and children included, 265 underwent transannular patch repair, and 76 received a right ventricular outflow tract patch. Postoperative transesophageal echocardiography was performed to evaluate for residual ventricular septal defects and to assess right ventricular outflow tract flow velocity. The necessity for reintervention was determined based on these intraoperative findings. Transthoracic echocardiography was assessed before discharge.

### Data collection

The covariates included preoperative patient characteristics (gender, age, comorbidities, O_2_ saturation, main pulmonary artery and pulmonary branches diameter, McGoon ratio), as well as intraoperative parameters (cardiopulmonary bypass time and aortic cross-clamp time). Postoperative variables included ultrasound results (pulmonary valve regurgitation, supravalvular flow velocity), chest drainage volume within 24 h, duration of mechanical ventilation, and length of ICU stay. Complications were also assessed, including secondary tracheal intubation, need for peritoneal dialysis, reoperation, ECMO usage, infection rate, and mortality rate. The McGoon ratio was calculated by the sum of left pulmonary artery (LPA) and right pulmonary artery (RPA) diameters divided by the aorta diameter at the diaphragm level.

### Statistical analysis

SPSS 27.0 software (IBM Corp., Armonk, NY, USA) was used to analyze the data. The study's population was first identified by demographic and preoperative clinical characteristics. Propensity score matching (PSM) was used to reduce baseline differences between two groups. Non-normally distributed data were expressed by the median [interquartile range (IQR)], and comparison between groups was performed by using the Mann–Whitney *U*-test. The mean ± standard deviation was used for counting data that obeyed or approximately obeyed a normal distribution, and the *t*-test was used for intergroup comparisons; Fisher's exact probability method or χ^2^ test was used for counting data. Differences were statistically significant with a *p* < 0.05.

## Results

There was a statistically significant difference in preoperative clinical data (age, weight, oxygen saturation, left and right pulmonary artery diameter, McGoon) between the two groups of patients (*p* < 0.05) (Table 2). To ensure the consistency of preoperative baseline data between the two groups, propensity score matching method was applied for 1:1 matching, resulting in two cohorts of 228 cases. After propensity matching analysis, there was no statistically significant difference in preoperative baseline data between the two groups (*p* *>* 0.05) (Table 3).

**Table 1:** Cannulation details for cardiopulmonary bypass in the two groups.

Weight (kg)	Minimally invasive group			Median sternotomy group		
	Superior Vena Cava catheter size, F	Inferior Vena Cava catheter size, F	Ascending Aorta catheter size, F	Superior Vena Cava catheter size, F	Inferior Vena Cava catheter size, F	Ascending Aorta catheter size, F
≤5 kg	10	12	6-8	10-12	12	8-10
5-10 kg	10-12	12	8-10	12	14	10-12
10-15 kg	12	12-14	10-12	12-14	14-16	12-14
15-20 kg	12-14	14	12-14	14-16	16-18	14-16
20-35 kg	14-16	14-16	14-16	16-20	18-22	16-20

**Table 2:** Clinical characteristics.

Group	Minimally invasive group (*n* = 147)	Median sternotomy group (*n* = 183)	*p*-value	*t*-value
Weight (kg)	7.57 ± 2.03	8.09 ± 1.79	0.031	0.869
Male (female)	89 (59)	108 (77)	0.746	0.105
Age (m)	8.50 ± 6.09	9.91 ± 5.67	0.015	0.459
comorbidity			0.696	1.439
TOF (only)	131	161		
ASD	11	11		
PDA	3	7		
Others	2	4		
Preoperative EF (%)	68.0 ± 4.4	67.4 ± 5.0	0.241	0.114
Preoperative O_2_ saturation (%)	90.0 ± 9.9	84.6 ± 11.7	0.000	0.10
McGoon ratio	1.77 ± 0.98	1.46 ± 0.39	0.000	0.114
Preoperative MPAd (mm)	9.7 ± 3.0	8.5 ± 2.9	0.000	0.305
RPAd (mm)	6.5 ± 1.7	5.9 ± 1.6	0.001	0.881
LPAd (mm)	6.5 ± 1.8	6.0 ± 1.8	0.013	0.955
RVOTd (mm)	7.8 ± 3.0	6.8 ± 2.9	0.001	0.131
Beta-blocker therapy (0.61%)	0 (0.00%)	2 (1.09%)	0.505	

TOF, tetralogy of Fallot; ASD, atrial septal defect; PDA, patent ductus arteriosus; others: tricuspid insufficiency; mitral insufficiency; MPAd, main pulmonary artery diameter; RPAd, right pulmonary artery diameter; LPAd, left pulmonary artery diameter; RVOTd, right ventricular outflow tract diameter.

**Table 3:** Clinical characteristics after propensity matching analysis.

Group	Minimally invasive group (*n* = 114)	Median sternotomy group (*n* = 114)	*p*-value	*t*-value
Weight (kg)	7.92 ± 2.01	7.74 ± 1.51	0.435	1.720
Male (female)	89 (59)	108 (77)	0.746	0.105
Age (m)	9.07 ± 6.65	8.41 ± 4.40	0.379	12.205
Comorbidity			0.615	1.800
F4 (only)	102	102		
ASD	9	6		
PDA	2	3		
Others	1	3		
CPB time (min)	107.4 ± 32.7	116.3 ± 37.0	0.055	1.464
Aortic cross-clamp time (min)	78.1 ± 24.7	82.6 ± 26.6	0.187	0.522
Preoperative EF (%)	67.5 ± 4.4	67.6 ± 5.2	0.847	1.323
Preoperative O_2_ saturation (%)	88.1 ± 10.5	88.0 ± 10.5	0.880	0.036
McGoon ratio	1.59 ± 0.37	1.59 ± 0.42	0.959	2.212
Preoperative MPAd (mm)	8.96 ± 2.64	9.05 ± 3.24	0.821	2.768
RPAd (mm)	6.21 ± 1.49	6.24 ± 1.70	0.898	2.980
LPAd (mm)	6.25 ± 1.63	6.29 ± 1.98	0.881	2.825
RVOTd (mm)	7.64 ± 3.01	7.26 ± 3.18	0.356	0.197
Beta-blocker therapy (0.88%)	0 (0.00%)	2 (1.7%)	0.498	

There was also no statistically significant difference between the two groups with respect to cardiopulmonary bypass time, aortic cross-clamp time, postoperative pulmonary valve regurgitation, and postoperative chest drainage volume within 24 h (*p* > 0.05). However, statistically significant differences were observed in duration of mechanical ventilation, ICU stay time, postoperative ejection fraction (EF) value, and postoperative pulmonary supravalvular flow velocity (*p* < 0.05) (Table 4). No statistically significant differences were found in mortality rate, secondary tracheal intubation, infection, or ECMO usage between the two groups (*p* > 0.05); however, the rate of peritoneal dialysis differed significantly between the groups (*p* < 0.05) (Table 4). The number of aortopulmonary collaterals occlusion in the median sternotomy group was higher than that in the minimally invasive group, and the difference was statistically significant (*p* < 0.05) (Table 4). Transthoracic echocardiography was assessed before discharge at the outpatient follow-up visit after 3 months. The results showed no obvious abnormality and no late death.

**Table 4:** Clinical information and complications between the two groups.

Groups	Minimally invasive group (*n* = 114)	Median sternotomy group (*n* = 114)	*p*-value	*t/Z*-value/χ^2^
Postoperative chest drainage volume within 24 h (ml)	58.2 ± 40.7	61.5 ± 35.7	0.506	0.694
Duration of ventilator tubing (h)	12.0 (7.0–26.3)	52.5 (22.8–100.5)	<0.001	14.425
ICU stay time (h)	70.0 (46.0–98.5)	114.5 (75.8–186.5)	<0.001	9.350
Blood usage within 24 h post-surgery (ml)	68.8 (0.0–136.3)	77.5 (0.0–156.3)	0.207	1.261
Postoperative pulmonary valve regurgitation (cm^2^)	0.61 ± 0.42	0.58 ± 0.36	0.671	2.117
Secondary tracheal intubation (4.82%)	7 (6.14%)	4 (3.51%)	0.354	0.860
Aortopulmonary collaterals occlusion (5.26%)	2 (1.75%)	10 (4.39%)	0.018	5.630
Peritoneal dialysis (7.46%)	4 (3.51%)	13 (11.4%)	0.023	5.149
Reoperation	0 (0.00%)	3 (2.63%)	0.247	
ECMO (2.19%)	1 (0.88%)	4 (3.51%)	0.369	
Infection (1.32%)	2 (1.75%)	1 (0.88%)	1.000	
Death (1.75%)	1 (0.88%)	3 (2.63%)	0.622	

CPB, cardiopulmonary bypass.

Data presented as median (interquartile range) or *n* (%).

## Discussion

Median sternotomy remains the gold standard cardiac surgical approach, offering optimal operative field exposure (9). Nevertheless, this technique inherently compromises sternal stability, induces significant surgical trauma, and results in suboptimal wound cosmesis. The conspicuous midline scar frequently causes psychological distress, particularly among pediatric and female patients (10). While advances in minimally invasive cardiac surgery have achieved technical maturity for infants and young children, current applications remain largely confined to simpler lesions including atrial septal defects, ventricular septal defects, and carefully selected endocardial cushion defects (11, 12). Compelling evidence confirms that right subaxillary thoracotomy represents a safe, feasible alternative associated with low mortality rates and satisfactory early-to-mid-term outcomes in elective TOF repair (13, 14). Building upon extensive institutional experience, our center has pioneered refined techniques for TOF correction via right axillary mini-thoracotomy in pediatric populations.

Technical Considerations:

1. For TOF cases with major aortopulmonary collateral arteries (MAPCAs), the resultant increased left heart venous return during cardioplegia may significantly obscure the surgical field (15). Our solution employing dual left atrial drainage catheters effectively controls this challenge. Preoperative multimodality imaging (echocardiography complemented by angiography or CT) is mandatory to evaluate collateral burden, thereby preventing catastrophic intraoperative conversion to full sternotomy (16). Concomitant patent ductus arteriosus can be reliably addressed through meticulous exposure and technique.
2. The extracorporeal circulation strategy is paramount in right axillary approaches. Given the higher complication rates associated with femoral cannulation in children <3 years (due to vessel caliber limitations), we advocate central cannulation (17). To overcome restricted space in the mini-thoracotomy, we utilize reduced-profile cannulae combined with vacuum-assisted venous drainage, achieving optimal flows without cannula-related complications (18, 19).

In the correction of children with TOF by the small incision on the right axilla, the operation is difficult and requires the experience of performing the correction of cardiac surgery with the small incision on the right axilla (20, 21). In our hospital, the simple atrial septal defect and ventricular septal defect in children have basically reached the level of performing the correction with the small incision on the right axilla, and the experience is rich. Compared with the median sternotomy group, there is no statistically significant difference in the extracorporeal circulation time and ascending aortic cross-clamp time in the minimally invasive group. After propensity matching analysis of preoperative pulmonary vascular development, age, weight, etc., it shows that the operation time is not delayed in the minimally invasive group.

Open-heart surgery and extracorporeal circulation in children with congenital heart disease can alter respiratory mechanics and impair alveolar diffusion, leading to compromised pulmonary function (22). The right axillary small incision approach necessitates partial lung compression to achieve surgical exposure, which poses a potential risk of pulmonary injury. To minimize this risk, we cover the surface of the right lung with wet gauze as a protective measure during surgery**.**

There were differences in preoperative weight, age, pulmonary vessel diameter, and McGoon ratio between the minimally invasive group and median sternotomy group, which were also the results of our preoperative selection. The McGoon ratio is associated with major adverse events after surgery in patients with tetralogy of Fallot (23), so the minimally invasive group is more suitable for children with better pulmonary vascular development and higher McGoon ratios. To make the prognosis of the two groups of children comparable, we conducted propensity score matching to compare the prognosis and complications between the two groups.

Compared with the median sternotomy group, the minimally invasive group demonstrated significantly shorter durations of postoperative mechanical ventilation and ICU stay, suggesting that the right axillary approach does not exacerbate pulmonary injury and is beneficial for postoperative recovery. The postoperative mortality rate was 2.63% in the median sternotomy group and 0.88% in the minimally invasive group. However, the difference in mortality rates between the two groups was not statistically significant. There were no significant differences between the two groups in the incidence of low cardiac output requiring ECMO support, infection, or secondary tracheal intubation. However, the incidence of peritoneal dialysis was significantly lower in the minimally invasive group than in the median sternotomy group. This finding may be attributable to patient selection, as children with prominent systemic-pulmonary collateral vessels were more likely to undergo median sternotomy. Our study also found that the number of aortopulmonary collaterals occlusion in the median sternotomy group was higher than that in the minimally invasive group, and the difference was statistically significant (*p* < 0.05). These patients tend to have a higher risk of right heart failure, systemic edema, and consequently, an increased likelihood of requiring postoperative peritoneal dialysis. On the other hand, children in the median sternotomy group had a relatively long cardiopulmonary bypass time, which may exacerbate cardiopulmonary bypass-associated renal injury. The specific reasons require further research and analysis.

## Conclusion

Our study demonstrated that minimally invasive correction through a right axillary incision in infants and young children with tetralogy of Fallot provided comparable safety outcomes to traditional median sternotomy, with significant advantages in shorter mechanical ventilation, ICU stay, and lower peritoneal dialysis rates. Given potential confounders such as anatomical variations and selection bias due to retrospective design, prospective randomized studies are warranted to conclusively validate these findings.

## Limitations

This was a retrospective single-center study using observational analysis. There was selection bias for the two surgical approaches. Particularly, for children with abundant systemic-pulmonary collaterals, we tend to prefer median sternotomy, which may cause a certain impact on the result.

## Data Availability

The raw data supporting the conclusions of this article will be made available by the authors without undue reservation.
